# Notification and Isolation

**Published:** 1898-08-13

**Authors:** 


### NOTIFICATION AND ISOLATION.

In opening the section of State Medicine at Edinburgh the president, Professor Sir Henry Littlejohn, said that one of the most melancholy things he had to mention was that there had been no great discovery in medicine or surgery but had been violently opposed by their own profession. The profession, to a man, in Edinburgh opposed the introduction of the Notification Act. The two Royal Colleges of Surgeons and Physicians instructed Parliamentary counsel to oppose the introduction of any such clause in the Bill which was being promoted by the Corporation of Edinburgh. Luckily, a kind Providence enabled them to catch their opponents napping. Something occurred; he did not know what, but their "omnibus" Bill was quietly shoved through Parliament, and the fatal clause was contained in it, and he had the satisfaction of intimating to his colleagues that this Act had been passed, and that it was his duty to carry it out in the most comfortable manner possible, and with the least offence to anyone. It had been a great success. They had a diminished mortality from all infectious disease. Edinburgh had the reputation in one or two volumes to be found in their library of being a fever city. They had had outbreaks. Let them think of 6,000 cases of typhoid in the course of a year! Medical schools suffered, for medical students died. That frightened parents, and Edinburgh got a bad reputation. Medical men in charge of the typhus also fell. They had an enormous mortality, both in the new and the old town. Rich and poor suffered alike, for in no part of the town had they any one quarter like a Ghetto. In the old town they had the University and their Law Courts, and if there was an epidemic in the old town, owing to the free communication between the southern part of the town and the new town, epidemics spread. That disease had been actually wiped out, chiefly owing to the improvements made in the rookeries and dens of the poor, and also specially by the Notification Act. In these upright "streets," accommodating 200 people, typhus spread like wildfire. Typhus epidemics, however, were a thing of the past, and no longer would Edinburgh be called a fever city so far as that scourge was concerned. They had sporadic cases, but they pounced upon them, and stamped out the disease. The strange thing in this connection was that they found the disease, typhus, to be as fatal as ever. He remembered Sir James Simpson, that man of genius, saying to him that diseases were undergoing cycles of changes, and were becoming less and less, that typhus would work itself out, and so on; but so far as their experience in Edinburgh was concerned typhus was as virulent as ever, and if it were not for their pouncing down upon it through the Notification Act, he believed Edinburgh would again acquire an unenviable notoriety. Referring to the City Hospital, Sir Henry said they had located in the centre of the poorest part of Edinburgh the hospital for infectious diseases, and that morning it contained over 300 cases; but, so far from that circumstance causing the disease to spread, it was remarkable that the immediate neighbourhood of the hospital showed a less amount of infectious disease than other parts of the city. He dissented from the view that there could be aërial communication of small-pox to any distance, and said he was perfectly sure that with the most ordinary care the disease would not spread.

